# Well-being and Anticipation for Future Positive Events: Evidences from an fMRI Study

**DOI:** 10.3389/fpsyg.2017.02199

**Published:** 2018-01-09

**Authors:** Yangmei Luo, Xuhai Chen, Senqing Qi, Xuqun You, Xiting Huang

**Affiliations:** ^1^Shaanxi Key Laboratory of Behavior and Cognitive Neuroscience, School of Psychology, Shaanxi Normal University, Xi’an, China; ^2^Key Laboratory of Cognition and Personality of Ministry of Education, School of Psychology, Southwest University, Chongqing, China; ^3^Ministry of Education Key Laboratory for Modern Teaching Technology, Shaanxi Normal University, Xi’an, China

**Keywords:** well-being, anticipation, positive affect, fMRI, medial prefrontal cortex

## Abstract

Anticipation for future confers great benefits to human well-being and mental health. However, previous work focus on how people’s well-being correlate with brain activities during perception of emotional stimuli, rather than anticipation for the future events. Here, the current study investigated how well-being relates to neural circuitry underlying the anticipating process of future desired events. Using event-related functional magnetic resonance imaging, 40 participants were scanned while they were performing an emotion anticipation task, in which they were instructed to anticipate the positive or neutral events. The results showed that bilateral medial prefrontal cortex (MPFC) were activated during anticipation for positive events relative to neutral events, and the enhanced brain activation in MPFC was associated with higher level of well-being. The findings suggest a neural mechanism by which the anticipation process to future desired events correlates to human well-being, which provide a future-oriented view on the neural sources of well-being.

## Introduction

As well-being is the central construct in positive psychology (Seligman and Csikszentmihalyi, 2000), substantial interest has been directed at delineating the sources of well-being. However, studies of well-being have been limited by the static, single point-in-time view (Gallagher et al., 2009). In particular, most of previous work focus on how people’s well-being correlate with brain activities during the perception of emotional events (e.g., van Reekum et al., 2007; Heller et al., 2013; Cunningham and Kirkland, 2014), rather than the anticipation for the upcoming events. For example, when emotional events were showed, happy people relative to their unhappy peers showed greater amygdala responses to positive stimuli (Cunningham and Kirkland, 2014), greater ventral anterior cingulate cortex (ACC) responses to negative stimuli (van Reekum et al., 2007). However, given that how people construct their future is a central organizing feature of perception, cognition, affect, memory, motivation, and action (Seligman et al., 2013), it is important to elucidate how anticipation for future contribute to people’s well-being, which can provide a dynamic, future-oriented view on the sources of well-being.

Anticipation, paying attention to the upcoming stimulus predicted by a contextual cue (Bermpohl et al., 2006), has important implications in human well-being and mental health. Anticipation confers important evolutionary benefits to human beings. Specifically, expecting the forthcoming events allow active preparations in cognitive, affective, and behavioral strategies (Grupe et al., 2013), which ensure survival in the changing and potential challenging environment (Gilbert and Wilson, 2007). Furthermore, the deficits of anticipation of future experience have been associated with extreme low levels of well-being, such as depression (MacLeod and Byrne, 1996; Abler et al., 2007) and anxiety (Nitschke et al., 2009; Boehme et al., 2014; Heitmann et al., 2014). Nitschke et al. (2009) found patients with generalized anxiety disorder differed from healthy control by showing hyperactivity in the amygdala when anticipating future aversive events. Together, it is necessary to investigate how neural circuitry underlying the anticipation for future events related to well-being.

Due to the fact that exaggerated negative anticipation contributes to the development and maintenance of emotional-related disorders (e.g., Abler et al., 2007; Nitschke et al., 2009; Aupperle et al., 2012; Boehme et al., 2014), most of previous studies focus on the neural circuitry during the anticipation of negative events (Nitschke et al., 2006; Herwig et al., 2007, 2010; Sarinopoulos et al., 2010; Yang et al., 2012; Grupe et al., 2013). However, it is argued that the anticipation of positive events is a key element of well-being (MacLeod and Conway, 2005), and thus deserving greater attention. On the one hand, the clinical literature shows there are distinct relationships between emotional disorders and positive or negative anticipation. For example, relative to healthy people, anxious people anticipated more negative future experiences, whereas depressed or parasuicidal people anticipated less positive future experiences, but did not anticipate more future negative events (MacLeod and Byrne, 1996; MacLeod et al., 1997). On the other hand, the studies in healthy samples demonstrate anticipating the positive events increases reward sensitivity (Gable et al., 2003), enhances the memory of positive stimuli (Crowell and Schmeichel, 2016), induces positive affect (Monfort et al., 2015), and relates to higher levels of well-being (MacLeod and Conway, 2005). A recent study found anticipating positive events (e.g., a funny cartoon) were a convenient and powerful way to induce positive emotion, which in turn improving stress coping (e.g., coping to a public speech) (Monfort et al., 2015). Taken together, these findings underline the distinctions between positive and negative anticipation and emphasize the contribution of the positive anticipation of future in people’s well-being (MacLeod and Conway, 2005).

Therefore, the current study focused on neural correlates of the relationship between well-being and anticipation for future desirable events. We intended to address this issue by employing the emotional anticipation task when participants were scanned by a functional magnetic resonance imaging (fMRI). Given that the amygdala and medial prefrontal cortex (MPFC) involve in the anticipating process (Nitschke et al., 2006; Bermpohl et al., 2008; Scherpiet et al., 2014), we hypothesized that the amygdala and MPFC would be activated during anticipating positive stimuli. Also, given the key role of amygdala in emotion processing (Davis and Whalen, 2001; Phelps and LeDoux, 2005; Sergerie et al., 2008) and different functional coupling between amygdala and prefrontal areas in various emotion processing (Lee et al., 2012; Diano et al., 2016, 2017; Di et al., 2017), we conducted psychophysiological interaction (PPI) analysis using left and right amygdala seeds. We hypothesized that positive anticipation would modulate functional coupling between amygdala and prefrontal cortex. Moreover, based on the close relationship between anticipation of future positive events and well-being (MacLeod and Conway, 2005; Monfort et al., 2015), we also hypothesized that the activation of these regions would correlate with the people’s well-being.

## Materials and Methods

### Participants

Forty right-handed participants (males/females, 15/25; mean age = 21.45 ± 1.74), with no history of neurological disorders and psychiatric disorders, were recruited among undergraduate and postgraduates populations. In accordance with the Declaration of Helsinki, written informed consent was obtained from all participants. The study protocol was approved by the Ethics Committee of the Southwest University. All participants were paid for their participation.

### Experimental Protocol and Stimuli

The stimuli were carefully selected from the Chinese Affective Picture System (CAPS) (Bai et al., 2005). We selected the stimuli from the CAPS instead of international affective picture system (Lang et al., 1997), because the CAPS is a collection of standardized photographic materials in the context of eastern culture to avoid cultural bias in emotional studies. However, the developing procedure of CAPS is identical to that of the international affective picture system (Bai et al., 2005). The stimuli from CAPS were successfully used to investigate the neural correlates of anticipation and perception of emotional events in previous studies (e.g., Yuan et al., 2007; Lin et al., 2012). Of these pictures, 52 depicted positive scenes (e.g., smiling kids, hugging, celebration, wedding, etc.), and 52 depicted neutral scenes (house appliances, pedestrians, a working man, women doing handwork, etc.). There were significant differences in valence (positive pictures: *M* = 6.97, *SD* = 0.21, max = 7.54, min = 6.50; neutral pictures: *M* = 5.39, *SD* = 0.25, max = 5.88, min = 4.68) (*t* = 36.47, *df* = 51, *p* < 0.001) and arousal (positive pictures: *M* = 6.07, *SD* = 0.42, max = 7.22, min = 5.29; neutral pictures: mean = 4.15, *SD* = 0.32, max = 4.80, min = 3.54) between positive pictures and neutral pictures (*t* = 28.09, *df* = 51, *p* < 0.001). There were no significant differences in valence and arousal among four fMRI runs for positive and neutral images, respectively (*p*s > 0.05). All the images were identical in size and resolution (433 pixel × 325 pixel, 72 pixels per inch). Mean luminance and contrast were balanced between two conditions.

**FIGURE 1:**
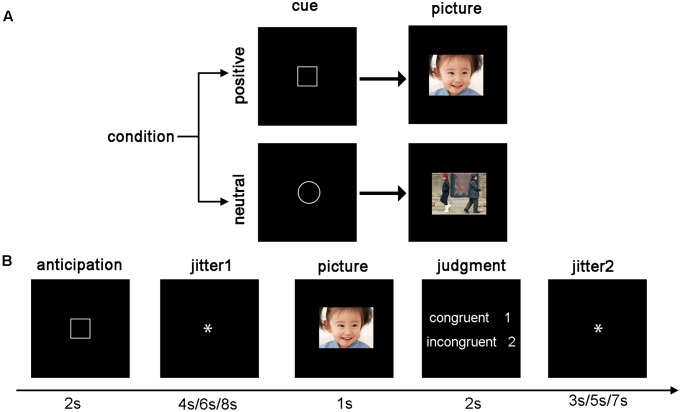
The fMRI experimental protocol. **(A)** The cue–picture pairings. The square signaled the following picture is positive, while the circle signaled that is neutral. **(B)** Example trials. Participants viewed a visual cue for 2 s, followed by a 4 s/6 s/8 s ISI, and a 1 s emotional image presentation. Then, participants made a congruent (incongruent) response depending on whether the cue was congruent with the valence of the picture. Lastly, another 3 s/5 s/7 s ISI were presented.

Participants were scanned while they completed an emotion anticipation task (**Figure 1**). Each trial started with a visual cue that signaled the following picture would be positive (“a square”) or neutral (“a circle”). The cue was presented for 2 s, followed by a 4 s/6 s/8 s jittered inter-stimulus interval (ISI), and a 1 s emotional image presentation. Then, participants were instructed to make a response to indicate whether the cue was congruent with the valence of the picture. If the cue and the valence of the picture were congruent (incongruent), participants responded with the number “1” (“2”) within 2-s. Participants were instructed about all cue–picture pairings prior to scanning. Lastly, another 3 s/5 s/7 s ISI were presented. Most of the cue–picture pairings were congruent, but we added two filler trials in each run (each condition has one, total eight trials), in which the cue and the valence of the images were incongruent, to keep the participants’ attention on the task. The filler trials were dropped due to their infrequency. There were 26 trials (12 positive, 12 neutral, and 2 filler trials presented in a pseudorandom order) in each of four functional runs lasting 6 min and 56 s. The total time of the experiment was about half an hour.

Following the scan, participants’ perceived well-being was assessed with 48-item Chinese Happiness Inventory (CHI) (Lu and Shih, 1997), composed of positive affect, negative affect, and life satisfaction. The CHI is composed of 20 “eastern” items deriving from Chinese culture and 28 “western” items from the Oxford Happiness Questionnaire (Argyle et al., 1989). It is a reliable measure of well-being in Chinese culture (e.g., Luo et al., 2014). Each item was presented in four incremental levels from unhappy to happy, numbered from 0 to 3. For example: I do not feel interested in being with family members (0); I seldom feel interested in being with family members (1); I often feel interested in being with family members (2); I always feel interested in being with family members (3). The higher score indicated a higher level of overall well-being. In our sample, a good reliability was observed (Cronbach’s α = 0.95).

### fMRI Data Acquisition

All images were collected on a 3.0-T scanner (Magnetom Trio, Siemens, Erlangen, Germany). Functional images were acquired using a single-shot, gradient-recalled echo planar imaging sequence (TR = 2000 ms, TE = 30 ms, flip angle = 90°, 32 axial slices, FOV = 192 cm × 192 cm, acquisition matrix = 64 × 64, slice thickness = 3 mm, without gap, voxel size = 3 mm × 3 mm × 4 mm). To minimize head motion, participants’ head were restricted with foam cushions. High-resolution T1-weighted anatomical images were also acquired in sagittal orientation using a 3D magnetization prepared rapid gradient-echo (MPRAGE) sequence (176 slices, TR = 1900 ms, TE = 2.53 ms, flip angle = 9°, resolution = 256 × 256, and voxel size = 1 mm × 1 mm × 1 mm) on each participant.

### fMRI Data Analysis

Data analysis was performed using FSL (FMRIB’s Software Library^1^) (Jenkinson et al., 2002; Smith et al., 2004). Pre-statistics processing consisted of motion correction using MCFLIRT (Jenkinson et al., 2002), slice-timing correction, non-brain removal using Brain Extraction Tool (BET; Smith, 2002), spatial smoothing (5 mm full-width at half maximum Gaussian kernel), and high-pass temporal filtering (Gaussian-weighted least-squares straight line fitting, with σ = 30.0 s).

For group analysis, we used the three-level approach in FEAT (FMRI Expert Analysis Tool, version 6.00), part of FSL. Firstly, a fixed-effects analysis modeled event-related responses for each run was computed, with the four explanatory variables (i.e., positive anticipation, neutral anticipation, positive perception, and neutral perception), and the six motion estimates as covariates of no-interest. The first two variables (i.e., positive anticipation and neutral anticipation) modeled the anticipation phase (cues onset to offset, 2 s duration, one regressor for positive cues, one regressor for neutral cues). Another two regressors (i.e., positive perception, neutral perception) modeled the perception phase (emotional images onset to offset, 1 s duration, one regressor for positive images, one regressor for neutral images). Each explanatory variable was convolved with a double gamma hemodynamic response function with a temporal derivative. According to our aim of this study, one contrast of interest was defined (positive anticipation > neutral anticipation). Functional volumes and first-level contrast images from this analysis were registered to corresponding structural volumes using boundary-based registration (BBR; Greve and Fischl, 2009), and then spatially registered to the standard space [Montreal Neurological Institute (MNI)] using 12 degrees of freedom with FMRIB’s Linear Image Registration Tool (FLIRT; Jenkinson et al., 2002).

A second-level analysis was performed combining the parameter estimates of the four runs for each participant, treating runs as a fixed effect. Then, these were inputs into the group level and a mixed-effects analysis was used to create group average maps for contrasts of interest. The *Z* statistical parametric maps were corrected for multiple comparisons using clusters determined by *Z* > 2.3 and a cluster significance of *p* = 0.05, based on Gaussian Random Field (GRF) theory (Worsley, 2001).

We conducted PPI analysis to examine whether functional connectivity between amygdala and prefrontal cortex was modulated by positive anticipation. MPFC, left and right amygdala were used as seeds in this analysis, respectively. MPFC were defined with a 6-mm radius sphere around the group peak activation voxel identified in the whole-brain analysis, and amygdala was defined with the Harvard–Oxford cortical atlas^2^, with the probability threshold set to 25%. The seeds were first transformed into functional space of each individual, and the time-series were extracted using individual mask. The first-level model included 11 regressors: psychological, physiological, PPI, two regressors in the perception phase, and six motion parameters. Positive and neutral trial durations comprised the psychological regressor, modeled with values 1 and -1, respectively, and convolved with double-gamma hemodynamic response function. The physiological regressor comprised the time-series for MPFC, the left or right amygdala, respectively. The PPI regressor modeled the interaction of the psychological regressor and the physiological regressor. Two regressors in the perception phase included positive perception and neutral perception. Group-level analysis was conducted using the same approach as the whole-brain analysis indicated above.

### Brain–Behavior Relationship

To assess whether neural regions involving in anticipation for positive events were correlated with well-being, we further extracted the percent signal change values from the significant cluster for positive > neutral contrast as outlined by Mumford (2007), and estimated a linear regression, with the percent signal changes values as a predictor, and the individual CHI score as a dependent variable.

## Results

### Behavioral Data

If the cue–picture pairings in the emotion anticipation task were congruent and participants were responded with the number “1” within 2 s, this trial was defined as “correct,” and vice versa. Paired *t*-tests were conducted on accuracy yielded no significant differences between the positive condition (*M* = 0.97, *SD* = 0.03) and neutral condition (*M* = 0.98, *SD* = 0.03) (*t* = -1.69, *df* = 39, *p* > 0.05), due to the simplicity of the task. Moreover, paired *t*-tests were conducted on reaction times (RTs) showed that the RTs for the positive condition (*M* = 512, *SD* = 131) were faster than that for the neutral condition (*M* = 579, *SD* = 146) (*t* = -5.90, *df* = 39, *p* < 0.001).

### Anticipation Activity for the Comparison of Positive to Neutral Condition

**FIGURE 2:**
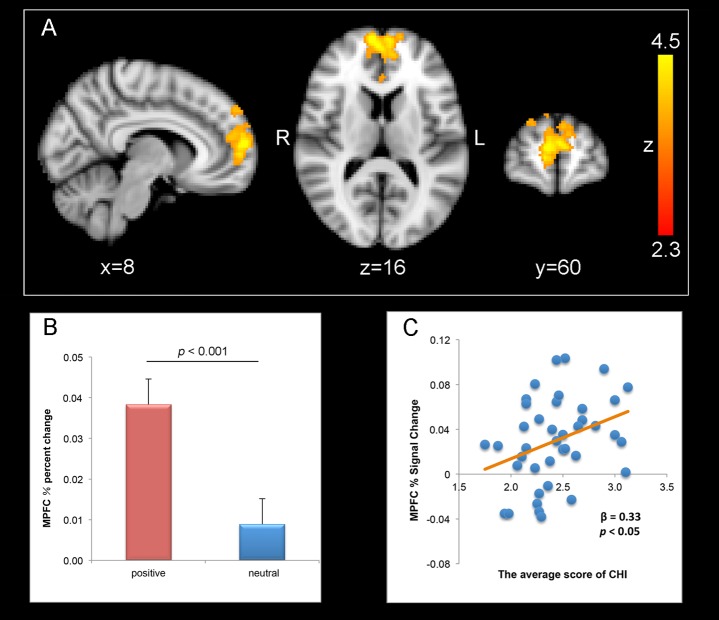
Correlation between well-being and MPFC activation for positive anticipation > neutral anticipation (thresholded at *z* > 2.3, *p* = 0.05, whole-brain corrected). **(A)** Bilateral MPFC (peak coordinates: *X* = 8, *Y* = 60, *Z* = 12) activation responding to positive anticipation > neutral anticipation. Numbers in the upper left corner of each image refer to the *x*-plane, *y*-plane, or *z*-plane coordinates of the MNI space (R, right and L, left). **(B)** Percent changes of MPFC in positive and neutral condition. Error bars represent the standard error of the mean. **(C)** Correlations between scores on the CHI and percent signal change values of the MPFC.

Whole-brain, cluster wise analysis for the contrast of the positive > neutral condition revealed that anticipating the positive stimuli relative to that for the neutral stimuli activated bilateral MPFC (peak MNI coordinates, 8, 60, 12; *Z*-score = 4.51; number of voxels = 1437, *p* < 0.001, GRF corrected) (**Figures 2A,B**).

The results of PPI analyses indicated MPFC (peak MNI coordinates, 10, 64, 10; *Z*-score = 3.33; number of voxels = 515, *p* < 0.001, GRF corrected) were activated in positive > neutral contrast. However, there aren’t brain areas that were functionally coupled with MPFC, left or right amygdala during positive than neutral condition, respectively.

### Brain–Behavior Relationship

The whole-brain analysis revealed that the MPFC were activated for positive anticipation relative to neutral anticipation. Thus, positive–neutral percent signal change values were extracted from the bilateral MPFC. And we then tested the relationship between the percent signal change of the MPFC on CHI by estimating a linear regression. The result showed that revealed the effects of percent signal change of the MPFC on CHI significant [β = 0.33, *t*(38) = 2.12, *p* < 0.05, *R*^2^= 0.11] (**Figure 2C**).

## Discussion

The present study investigated how brain activities during anticipation for positive events were associated with individual difference of well-being. Participants performed an emotion anticipation task in which they were required to anticipate the positive events or neutral events and then made judgments whether the cue and the following affective stimuli was congruent or not. It was found that people made faster judgments in the positive condition than that in the neutral condition, which may indicate people’s greater motivation to positive stimuli relative to neutral stimuli. More importantly, the bilateral MPFC were activated in response to the positive cues relative to neutral cues, and the MPFC activations were positively correlated with people’s perceived well-being.

We found that the bilateral MPFC were involved in anticipating the positive stimuli relative to the neutral stimuli. MPFC is highly implicated in a variety of cognitions, not only self-referential processing (Gusnard et al., 2001; Northoff et al., 2006) and mentalizing (Amodio and Frith, 2006), but also the emotion and reward processing (Ochsner and Gross, 2005; Kringelbach and Berridge, 2009; Etkin et al., 2011). In particular, MPFC is not only involved in emotional perception (Costa et al., 2010), but also in emotional anticipation (Sharot et al., 2007; Bermpohl et al., 2008; D’Argembeau et al., 2010). Specifically, D’Argembeau et al. (2010) found anticipating personal future goals elicited stronger activation in MPFC relative to non-personal future events. Therefore, our results were consistent with previous studies. Taken together, we speculated that the activation of MPFC might represent the anticipatory pleasures during expecting the future positive events.

More interestingly, we found that the MPFC activation during anticipating the positive events were positively correlated with individual difference of well-being. The results are consistent with previous neural evidence, which found the cues signaling emotional events activated MPFC and the activations were positively correlated with novelty-seeking (Bermpohl et al., 2008), a personality trait characterized by seeking new and potential pleasures (Kashdan and Steger, 2007). Previous work focuses on the neural circuitry underlying hedonic enjoyment of consumption of positive stimuli associating with well-being (e.g., Epstein et al., 2006; van Reekum et al., 2007; Heller et al., 2013; Cunningham and Kirkland, 2014). However, except the emotional perception, our study found neural activation during emotional anticipation was related to people’s well-being. The findings extended past work by providing a dynamic, future-oriented view on the well-being.

Furthermore, our results were consistent with the goal theory of well-being (Carver and Scheier, 1990; Diener et al., 1999). This framework proposed that having and progressing goals confer benefits to well-being (Carver and Scheier, 1990; Diener et al., 1999). The benefits to well-being are not only from goal achievement, but also from the strong positive anticipation element in goal progress (MacLeod and Conway, 2005). Goals and goal progress is possible to enhance people’s well-being through positive anticipation (MacLeod and Conway, 2005). The results were consistent with our previous resting state work, which indicated the happy people relative to their unhappy peers showed increased local functional connectivity within MPFC (Luo et al., 2014). Combining the resting and task-based neuroimaging results, we speculated that people with higher level of well-being may derive more pleasure from anticipating future positive events, which may relative to the desirable goals. In this regard, our results may provide the neural evidence for the goal theory of well-being.

Anticipation for future is a process of emotion regulation (Erk et al., 2006; Grupe et al., 2013). Thus, the neuroimaging results can also be interpreted from the perspective of emotion regulation. Anticipating the future allows allocating cognitive and emotional resources and planning behavior strategies to cope the upcoming events (Erk et al., 2006; Grupe et al., 2013). For example, anticipating positive events can induce positive emotion to cope the social stress (e.g., Monfort et al., 2015). Previous findings suggest that MPFC and ACC are highly implicated in emotion regulation (Phillips et al., 2008; Etkin et al., 2015). Specifically, MPFC and ACC are thought to be part of model-free emotion regulation system, in which MPFC and ACC encoding the experience-dependent value of regulatory actions, which modulate activity in emotional-reactivity regions, such as the amygdala, insula, dorsal ACC, and periaqueductal gray (Etkin et al., 2015). The MPFC activation during the anticipating future positive events in our study may indicate people felt more positive emotion when anticipating positive future, which in turn enhanced people’s well-being. In this perspective, our results were consistent with Etkin et al.’s (2015) framework.

However, we did not observe the functional connectivity between MPFC and other emotion-related areas, such as the amygdala. Also, we did not observe amygdala activation during positive anticipation relative to neutral anticipation. Our results were inconsistent with prior studies which show that amygdala is highly involved in emotional perception (e.g., Sergerie et al., 2008; Cunningham and Kirkland, 2014) and has different coupling pattern with other areas during different tasks of emotion processing (Lee et al., 2012; Diano et al., 2016, 2017; Di et al., 2017). Reviewing the literature of neural correlates of emotion anticipation, we found amygdala was consistently activated during anticipation of aversive events, such as negative picture (e.g., Ueda et al., 2003; Erk et al., 2006; Nitschke et al., 2006) and pain (e.g., Wager et al., 2004), but there is little evidence that amygdala was involved in positive anticipation (e.g., Ueda et al., 2003; Scherpiet et al., 2014). One reason that the lack of significant results in the amygdala in our study could be that amygdala was more involved in negative anticipation, but the task we employed didn’t include anticipation of negative events. Our results might indicate that amygdala was involved in perception phase of positive emotion events (e.g., Cunningham and Kirkland, 2014), but it didn’t be involved in the anticipation phase. However, the role of amygdala in the emotion anticipation needs further investigation.

Although we provide evidence that MPFC activation during positive anticipation can predict the levels of well-being, several limits and future directions of this study need to be considered. Firstly, the cross-section design hinders the conclusion of the causal relationships between the brain activity and well-being. It will be of merit for future work using neuroimaging modulation technique, such as transcranial magnetic stimulation (TMS) and transcranial direct current stimulation (tDCS), to alter brain activity and find the causal relationship. Secondly, we did not find the amygdala activation in positive anticipation, but the previous study found amygdala was activated when positive stimuli were presented (Cunningham and Kirkland, 2014). This may indicate the distinct brain regions involved in emotional perception and anticipation. Nevertheless, whether the amygdala is activated in positive anticipation needs further investigation. Thirdly, we speculated the psychological processes during anticipation in this study. However, future studies may be beneficial to collect the online self-report of the motivation to the hedonic cue when people were anticipating. Fourthly, the contents of pictures used in positive and neutral condition weren’t rigidly matched in the current study. Although we tried to select pictures depicted people’s activities in both conditions, the content of the stimuli in both conditions were not rigidly matched. The content difference of the pictures between positive and neutral condition may confound the neural activation results. For example, amygdala may respond to human faces or bodies (Van den Stock et al., 2014; Wang et al., 2014) but not to household appliances. However, this may not a fatal factor that confounded our results, because we are interest in the anticipation phase of emotion instead of the perception phase. We rigidly matched the physical properties of the cues between two conditions. But the contents of pictures from both conditions should be rigidly matched in future studies. Lastly, our study did not include the anticipation for negative events to control for potentially confounding factors. However, given the negative psychopathological consequences resulting from exaggerated negative anticipation (Grupe and Nitschke, 2013; Heitmann et al., 2014), future studies examining the association between well-being and neural underpinning of anticipation to the negative emotion may be interesting.

## Conclusion

In this study, we used emotional anticipation task to investigate how the neural underpinning during anticipating to future positive events was associated with individual difference of well-being. The results showed that bilateral MPFC was activated during anticipation for positive events relative to neutral events, and MPFC activities were positively correlated the levels of well-being. The findings may be consistent with the goal theory of well-being and provide the dynamic, future-oriented view on well-being.

## Author Contributions

YL and XH conceived and designed the experiments. YL performed the experiments. YL and XC analyzed the data. YL, XC, SQ, XY, and XH wrote the paper. All authors contributed to and have approved the final manuscript.

## Conflict of Interest Statement

The authors declare that the research was conducted in the absence of any commercial or financial relationships that could be construed as a potential conflict of interest. The reviewer MD and handling Editor declared their shared affiliation.

## Footnotes

1 www.fmrib.ox.ac.uk/fsl

2 https://fsl.fmrib.ox.ac.uk/fsl/fslwiki/Atlases
